# Erythropoietin Levels Increase during Cerebral Malaria and Correlate with Heme, Interleukin-10 and Tumor Necrosis Factor-Alpha in India

**DOI:** 10.1371/journal.pone.0158420

**Published:** 2016-07-21

**Authors:** Esther Dalko, Nicolas Tchitchek, Laurent Pays, Fabien Herbert, Pierre-André Cazenave, Balachandran Ravindran, Shobhona Sharma, Serge Nataf, Bidyut Das, Sylviane Pied

**Affiliations:** 1 Centre for Infection and Immunity of Lille, INSERM U1019, CNRS UMR 8204, Université Lille Nord de France, Institut Pasteur de Lille, Lille 59019, France; 2 CEA, DSV/iMETI, Immunology of viral infections and autoimmune diseases research unit, UMR1184, IDMIT infrastructure, Fontenay-aux-Roses, France; 3 Lyon 1 University, CarMeN Laboratory, INSERM U-1060, INRA USC-1235, 69921, Oullins, France; Banque de Tissus et de Cellules des Hospices Civils de Lyon, Hôpital Edouard Herriot, Lyon, France; 4 Institute of Life Sciences, Bhubaneswar, Odisha, India; 5 Department of Biological Sciences, Tata Institute of Fundamental Research, Mumbai, Maharashtra 400005, India; 6 SCB Medical College, Cuttack, Odisha 753007, India; Centro de Pesquisa Rene Rachou/Fundação Oswaldo Cruz (Fiocruz-Minas), BRAZIL

## Abstract

Cerebral malaria (CM) caused by *Plasmodium falciparum* parasites often leads to the death of infected patients or to persisting neurological sequelae despite anti-parasitic treatments. Erythropoietin (EPO) was recently suggested as a potential adjunctive treatment for CM. However diverging results were obtained in patients from Sub-Saharan countries infected with *P*. *falciparum*. In this study, we measured EPO levels in the plasma of well-defined groups of *P*. *falciparum*-infected patients, from the state of Odisha in India, with mild malaria (MM), CM, or severe non-CM (NCM). EPO levels were then correlated with biological parameters, including parasite biomass, heme, tumor necrosis factor (TNF)-α, interleukin (IL)-10, interferon gamma-induced protein (IP)-10, and monocyte chemoattractant protein (MCP)-1 plasma concentrations by Spearman’s rank and multiple correlation analyses. We found a significant increase in EPO levels with malaria severity degree, and more specifically during fatal CM. In addition, EPO levels were also found correlated positively with heme, TNF-α, IL-10, IP-10 and MCP-1 during CM. We also found a significant multivariate correlation between EPO, TNF-α, IL-10, IP-10 MCP-1 and heme, suggesting an association of EPO with a network of immune factors in CM patients. The contradictory levels of circulating EPO reported in CM patients in India when compared to Africa highlights the need for the optimization of adjunctive treatments according to the targeted population.

## Introduction

Malaria eradication is a worldwide public health priority. Despite tremendous efforts, children and adults still die from this plague every year due to severe disease manifestations. Cerebral malaria (CM) is the deadliest complication, and as reported by the World Health Organization it is most of the time caused by *Plasmodium falciparum* infections [1]. The pathophysiology of CM is complex and far from being completely understood. It involves brain inflammation triggered by the sequestration of parasitized erythrocytes, cell death and infiltrating immune cells in the central nervous system, as well as the systemic production of pro-inflammatory cytokines [2]. In 18% of the cases, patients die despite receiving anti-parasitic treatment, and surviving patients often suffer from neurological sequelae [2,3]. Thus, there is an important need in finding appropriate adjunctive treatments against CM.

In this perspective, administration of erythropoietin (EPO) has recently raised interest in the treatment of neurodegenerative diseases, including CM. EPO, first described as a hematopoietic hormone, is principally produced by peritubular capillary endothelial cells of the kidney in response to hypoxia. Upon binding to its homodimeric receptor, EPO-R, EPO stimulates the expression of transcription factors such as Bcl-X_L_ that further stimulates the maturation of erythroid progenitors [4]. Interestingly, the *in vitro* analysis of human brain cells have shown that astrocytes also constitutively produce low levels of EPO, which vary in response to cytokines [5]. In addition, neurons, glial cells, and endothelial cells, express EPO-R, suggesting a plausible localized impact of this hormone on the central nervous system [5,6]. EPO may exert extra-hematopoietic functions in the brain, diminishing the production of pro-inflammatory cytokines and preventing neuronal cell death, which appear to limit brain damage and neurological sequelae [7–9].

Several studies have suggested that EPO plays a pivotal role in CM by exerting protective effects within the central nervous system. Elevated EPO levels were found in plasma of children with severe *P*. *falciparum* malaria in Mozambique, including severe malarial anemia, but not within the CM group [10]. Furthermore, high levels of endogenous plasma EPO were associated with reduced risk of developing acute neurological sequelae after CM in Kenya [11]. EPO was also shown to efficiently prevent experimental CM during *P*. *berghei* ANKA infection of C57BL/6 and CBA/J mice [12–14]. It was likely that the beneficial impact of recombinant human EPO (rhEPO) on CM was concurrent to decreased production of the pro-inflammatory cytokines interferon (IFN)-γ and tumor necrosis factor (TNF)-α, and decreased recruitment of T lymphocytes within the brain [12–14]. These results were the basis for conducting a safety clinical trial in Mali, using 1,500 IU/kg rhEPO as adjunctive treatment during three days after admission of the patients, and combined to quinine for CM [15]. However, a very recent study published by Shabani et al., reported elevated plasma EPO levels in CM cases in children with prolonged coma and increased mortality [16]. In addition, considerable uncertainty still remains regarding the use of rhEPO as a neuroprotective strategy for the treatment of other neurodegenerative disease such as acute stroke. In fact, rhEPO given at the admission of the patients was first associated with smaller lesion size and a better functional recovery of the patients compared to the placebo [17], but a later clinical trial with a higher number of patients has reported deleterious outcomes for the survival of acute stroke patients [18].

In light of these discrepancies and the absence of studies concerning populations with different age and environmental factors, we have investigated EPO levels in groups of *P*. *falciparum*-infected patients with mild malaria (MM), severe non-cerebral malaria (NCM), or CM from the state of Odisha, India. In 2010, approximately 85% of the Indian population lived in malaria-endemic areas, representing 27% of malaria cases outside Africa [19]. Unlike in Sub-Saharan Africa, mainly adults develop severe malaria in India, with very heterogeneous clinical pictures that are usually not associated with anemia [20–22]. We addressed the relationship between EPO levels and the clinical severity in *P*. *falciparum*-infected adults. In addition, we examined the association of EPO levels with biological parameters associated to the lysis of red blood cells [hemoglobin, *P*. *falciparum* histidine-rich protein-2 (PfHRP-2), hemopexin and total heme levels], and to pro- and anti-inflammatory cytokine associated to heme levels and involved in CM pathophysiology.

## Materials and Methods

### Ethics

This study was conducted according to the guidelines set out in the Declaration of Helsinki. The study was approved by the Institutional Human Ethics Committee of SCB Medical College, Cuttack, India, and the Institutional Review Board of all three collaborating institutes: (i) the Institute of Life Sciences, Bhubaneswar, India, (ii) the Tata Institute of Fundamental Research, Mumbai, India, and (iii) Institut Pasteur de Lille, Lille, France. The National Health Office Ethics committee in India approved the study design. All blood samples were collected after obtaining written consent from participants or, in the case of comatose patients, accompanying relatives.

### Study site and participants

This study is a retrospective analysis of 130 patients admitted at the Department of Medicine of the Sriram Chandra Bhanj Medical College & Hospital (SCB Medical College), Cuttack, Odisha in India, between 2008 and 2011. We have already analyzed these subjects in other studies [20,21]. All patients of age ≥ 15 years, with a history of fever and with evidence of altered sensorium, jaundice, oliguria, respiratory distress, shock, and/or bleeding diathesis were screened for *Plasmodium* infection by Giemsa blood smears and an immune chromatography test (SD Bio Standard Diagnostics, India). As described earlier, patients who were positive by immune chromatography test but negative by blood smears were subjected to nested PCR [23]. Only *P*. *falciparum*-infected patients were included; all *P*. *vivax* co-infections were excluded from the study.

Following confirmation of *P*. *falciparum* infection, patients were categorized into distinct clinical groups; MM patients only developed fever, and severe malaria (SM) patients were defined as those having at least one laboratory or clinical feature of severe malaria reported by the WHO [24]. Further categorization of SM patients into two groups, CM and NCM, was based on distinct clinical features. CM were defined as patients with a Glasgow Come Score (GCS) ≤ 10 after exclusion of other causes of encephalopathy such as encephalitis, meningitis and metabolic encephalopathy by biochemical investigations in the cerebrospinal fluid, and NCM patients were SM patients without cerebral involvement. All severe anemia cases, defined by the WHO as hemoglobin levels < 7 g/dL for adults [1], were excluded from the study. Endemic controls (EC) of identical ethnicity and coming from a similar geographical background were enrolled from among patients’ relatives (n = 25), and did not report a history of clinical malaria in the five years preceding our study.

### Blood collection and assessment of biological parameters

Peripheral venous blood (5 mL) was collected on day 0, before treatment, by vein puncture in sterile EDTA tubes. Plasma was obtained by centrifuging blood samples at 4500g for 15 min, and was stored at −80°C until further use. Complete blood count was performed with 2 mL of blood collected in EDTA and using an automated cell counter (Sysmex XT-2000 Hematology Analyzer, Japan). All biological parameters described earlier were measured in peripheral venous blood including blood sugar, electrolytes, creatinine, transaminases and lactate levels [20,21]. Those with evidence of bacterial (urinary tract infection, sepsis) and viral (hepatitis A, E, B, C) infections were excluded from the study, as well as patients affected with metabolic diseases, including diabetes type 2.

### Quantification of plasma levels of *P*. *falciparum* histidine rich protein-2

PfHRP-2 levels were measured in plasma by ELISA, and as described previously [25]. Briefly, an anti-PfHRP-2 IgM (MPFM-55A; Santa Cruz Biotechnology) was used as the capture antibody; an anti-PfHRP-2 IgG1 was used as a detection antibody (MPFG-55; Santa Cruz Biotechnology), and an HRP-coupled monoclonal anti-IgG1 antibody (SB77e; Abcam) was used as the secondary antibody for detection. A standard curve was added in duplicate to each plate, and it was obtained through serial dilutions of a plasma sample from a previously described cohort with known parasitemia [26]. Samples out of the range of detection were more or less diluted. The lower limit of detection was 0.5 arbitrary units (AU).

### Determination of EPO, total heme, hemopexin and heme oxygenase (HO)-1 levels in plasma

Plasma levels of EPO, hemopexin and HO-1 were measured by ELISA using, respectively, the Human EPO Platinum ELISA (eBioscience), the Human Hemopexin ELISA kit (Abcam), and the Human HO-1 ELISA kit (Enzo Life Sciences), and according to the manufacturer’s instructions. Total heme levels were measured in plasma using the QuantiChrom Heme Assay kit (BioAssay Systems). The colorimetric reaction was measured at 405 nm.

### Assay of cytokines

TNF-α, interleukin (IL)-10, interferon gamma-induced protein (IP)-10, and monocyte chemoattractant protein (MCP)-1 levels were measured in plasma using the MILLIPLEX MAP multiplex assay kit (Millipore), as described previously [20].

### Statistical analysis

**Fig 1:**
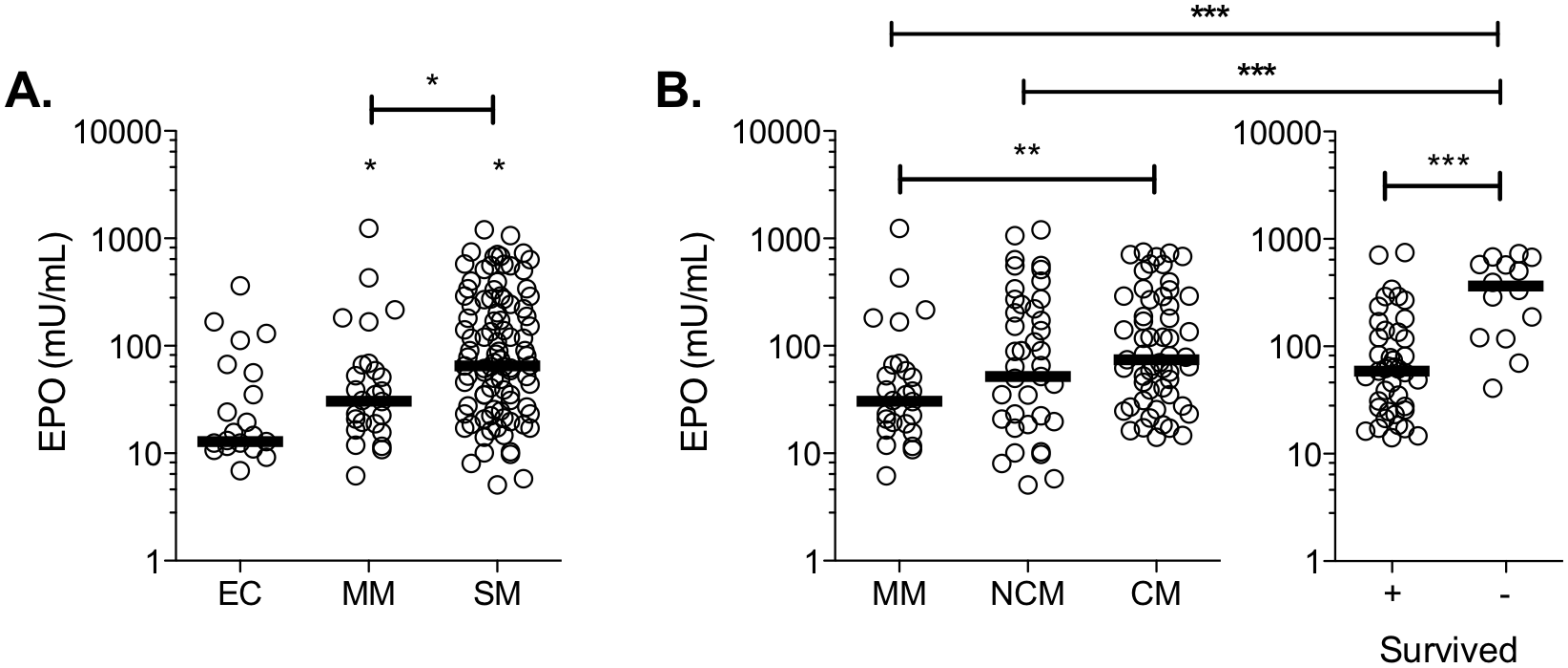
Erythropoietin levels increase during cerebral malaria and with severity of the infection. (A) Erythropoietin (EPO) levels were measured in the plasma of endemic controls (EC), mild malaria (MM) and severe malaria (SM) patients, and (B) in the SM sub-groups of non-cerebral malaria (NCM) and cerebral malaria (CM). (C) EPO levels were compared between CM patients who survived and those who did not. Significant differences with the EC group or between selected groups are indicated as the following: *p ≤ 0.05; **p ≤ 0.01; ***p ≤ 0.001.

Statistical analyses of erythropoietin levels (EPO) presented in Fig 1 have been performed using GraphPad Prism Software (version 7) and R [27]. Data have been tested for variance equality using the Levene test, and for non-normality using D’Agostino-Pearson omnibus normality tests (*p*-values < 0.0001). The Kruskal-Wallis test showed that the distribution of ranks was significantly different in some groups (*p*-value < 0.0001). Sample groups were then compared using the non-parametric Mann-Whitney test and significant differences were defined as comparisons with a *p*-value below 0.05.

The correlation between EPO plasma levels and biological parameters were determined using the Spearman coefficient of correlation. Multiple correlations analyses have been calculated using linear fitting models. Computations of the linear models have been performed using the ‘lm’ function of the native ‘stat’ package in R [27]. For each regression model used in this paper, the estimated value, standard error, *t*-value and *p*-value are provided for each coefficient. Moreover for each regression model, the Spearman coefficient of correlation between data and fitted data is provided with its confident interval and the associated p-value. A *p*-value < 0.05 was considered as significant.

The GeneMANIA Cytoscape plugin (v3.4.0) was used to generate 2 distinct co-expression networks from the clinical, biological and cytokine parameters obtained in severe NCM and CM patients [28]. The severe NCM network was composed of 532 interactions and the CM network of 628 interactions. A supervised clustering was then performed to identify the top ten parameters that co-regulated with EPO levels. For presentation purposes, nodes with the highest level of connectivity were assigned a different shape using Cytoscape (v3.2.1), an open-source network visualization software [29].

## Results

### Baseline characteristics

A total of 130 patients were included in this study: 28 MM, 45 NCM and 57 CM patients. The strong male bias is in agreement with studies previously reported in India [22,30]. There were no differences in patients’ age, and hemoglobin levels between MM, NCM and CM groups (Table 1). PfHRP-2 levels were shown to be good indicators of total parasite biomass [31], and they were significantly higher in the plasma of NCM and CM compared to MM patients (Table 1).

**Table 1:** Baseline characteristics.

	MM	NCM	CM	All
**No.**	28	45	57	130
**Age (year)** ^#^	30 (24–35)	28 (23–41)	30 (20–43)	30 (22–41)
**% Male**	60.7	77.8	86.0	77.7
**Hb (g/dL)** ^#^	10.6 (9.9–12.3)	10.0 (9.0–12.0)	10.0 (8.6–11.3)	10.2 (9.0–12.0)
**PfHRP-2 (AU)** ^#^	11.9 (1.1–25.3)	22.0 (8.3–38.5)*	29.7 (21.2–47.8)***	23.2 (9.7–40.8)

^#^ Values are medians (interquartile ranges).

* MM vs. NCM: *p*-value < 0.05.

*** MM vs. CM: *p*-value < 0.001.

### EPO levels increase during CM

To investigate if EPO levels were associated with a “protective phenotype” against CM in Odisha, EPO levels were determined in the plasma of *P*. *falciparum*-infected patients and EC. To eliminate a possible bias resulting from low hemoglobin levels, patients with severe anemia (Hb < 7 g/dL) were excluded from the study [1]. Surprisingly, we found that EPO levels were significantly higher in the plasma of SM patients compared to EC, and MM (Fig 1A). More specifically, only CM patients had significantly higher plasma EPO compared to MM patients (Fig 1B, left panel). EPO levels were not significantly different between MM and NCM patients, as well as between CM and NCM. In our groups of patients, only those developing neurological outcomes died, and we show a 6.7-fold increase in the median levels of EPO among the group of CM patients who died (n = 16) compared to CM survivors (n = 41) (Fig 1B, right panel); hemoglobin levels were not significantly different between these two groups (*p*-value = 0.3559). In addition, EPO levels were significantly higher in plasma of CM non-survivors compared to NCM and MM patients (Fig 1B, right panel), suggesting that high levels of EPO are associated with lethality in Odisha. It is to note that age, sex, PfHRP-2 levels and hemopexin levels were independently associated with mortality.

### EPO levels correlated with biological parameters

During blood stage malaria, EPO production by the kidney increases to counteract the loss of red blood cells. Considering that in our groups of malaria-infected patients hemoglobin levels correlated significantly and negatively with PfHRP-2 plasma levels (R = -0.217; *p*-value = 0.0013), we investigated correlations between EPO and hemoglobin, PfHRP-2, total heme and hemopexin plasma levels to further characterize the conditions associated to enhanced EPO levels. When considering all *P*. *falciparum*-infected patients, EPO correlated significantly with hemoglobin and PfHRP-2 plasma levels, with a negative and a positive factor, respectively. In addition, EPO plasma levels correlated with hemoglobin for MM and CM patients, but not for the NCM ones. A correlation between EPO and PfHRP-2 levels was found for both NCM and CM (Table 2). We have previously reported that total heme and hemopexin levels are good indicators for free heme levels [21]. Hemopexin levels correlated negatively with EPO when analyzing all *P*. *falciparum-*infected patients, as well as both SM groups, the NCM and the CM group. EPO correlated positively with total heme only for CM patients (Table 2). As shown by multiple correlation analyses, EPO levels correlated significantly to both sets of data: [hemoglobin + PfHRP-2 + hemopexin + total heme] (R = 0.50; CI (0.359–0.618); *p*-value = 0.0001), and [hemopexin + total heme] (R = 0.45; CI (0.302–0.577); *p*-value = 0.0001) (Tables 3 and 4). According to our analyses, mainly hemoglobin and hemopexin levels are likely to associate with EPO levels as suggested by their low Pr (>|t|), and with a negative factor of the estimated coefficient.

**Table 2:** Correlation of erythropoietin plasma levels, with biological parameters.

	MM	NCM	CM	All
**Hemoglobin**				
R	-0.42	-0.25	-0.35	-0.34
*p*-value	0.0421	0.1070	0.0118	0.0002
**PfHRP-2**				
R	0.32	0.38	0.27	0.41
*p*-value	0.0922	0.0127	0.0419	< 0.0001
**Hemopexin**				
R	-0.22	-0.41	-0.41	-0.44
*p*-value	0.2534	0.0069	0.0024	< 0.0001
**Total heme**				
R	-0.04	0.03	0.30	0.17
*p*-value	0.8500	0.8532	0.0209	0.0526

R: Spearman coefficient of correlation.

**Table 3:** Multiple correlation analyses of [EPO] with [hemoglobin+PfHRP-2 + hemopexin + total heme].

	Estimate	Std. Error	*t*-value	Pr (>|t|)
(Intercept)	456.3511	126.2689	3.614	0.0005
Hemoglobin	-25.6799	11.6281	-2.208	0.0292
PfHRP-2	0.8461	0.7130	1.187	0.2378
Hemopexin	-0.3550	0.1464	-2.425	0.0169
Total heme	0.0103	0.3874	0.027	0.9788

The estimated coefficient (Estimate) corresponds to the fitted variable value, the standard error (Std. Error) indicates the variability of the estimated coefficient, the t-value (*t*-value) measures if the estimated coefficient for this variable is meaningful for the model, and the variable p-value (Pr (>|t|)) indicates the probability that the variable is not relevant in the model. R = 0.50; CI (0.359–0.618); *p*-value = 0.0001.

**Table 4:** Multiple correlation analyses of [EPO] with [hemopexin + total heme].

	Estimate	Std. Error	*t-*value	Pr (>|t|)
(Intercept)	227.5893	41.9219	5.429	2.77e-07
Hemopexin	-0.4708	0.1268	-3.713	0.0003
Total heme	0.1330	0.3660	0.363	0.7169

The estimated coefficient (Estimate) corresponds to the fitted variable value, the standard error (Std. Error) indicates the variability of the estimated coefficient, the *t*-value measures whether or not the estimated coefficient for this variable is meaningful for the model, and the variable *p*-value (Pr (>|t|)) indicates the probability that the variable is not relevant in the model. R = 0.45 CI (0.302–0.577); *p*-value = 0.0001.

Given our findings on the heme/hemopexin balance that influences cytokine profiles during severe malaria [32], we first investigated a possible correlation between EPO, TNF-α, IL-10, IP-10 and MCP-1 plasma levels. These cytokines have been shown to contribute to the pathophysiology of human CM in India [20,22]. All four cytokines were positively correlated to EPO levels during malaria, as well as to CM cases. EPO levels were not correlated to IP-10 and MCP-1 levels during MM (Table 5). Interestingly, the set of data [EPO + hemopexin + total heme] correlated significantly to [TNF-α + IL-10 + IP-10 + MCP-1] during infection, and more specifically for the CM, and NCM groups, but not for MM patients (Tables 6–9). During *P*. *falciparum* infections, IL-10 and MCP-1 associated positively to [EPO + hemopexin + total heme] as suggested by their respective estimated coefficient 0.0616 and 0.0602 that associated to a low variable *p*-value (Table 9). IP-10 was also shown to contribute to the statistical model during CM, but associated to [EPO + hemopexin + total heme] with a negative factor as indicated by the estimated coefficient of -0.0198 (Table 8).

**Table 5:** Correlation of EPO plasma levels, with cytokines.

	MM	NCM	CM	All
**TNF-α**				
R	0.42	0.13	0.42	0.32
*p*-value	0.0355	0.4046	0.0021	0.0005
**IL-10**				
R	0.40	0.57	0.51	0.52
*p*-value	0.0479	0.0003	0.0002	< 0.0001
**IP-10**				
R	0.10	0.64	0.35	0.49
*p*-value	0.6117	< 0.0001	0.0111	< 0.0001
**MCP-1**				
R	0.05	0.58	0.50	0.50
*p*-value	0.8042	0.0002	0.0002	< 0.0001

R: correlation coefficient.

**Table 6:** Multiple correlation analyses of [EPO + hemopexin + total heme] with [TNF-α + IL-10 + IP-10 + MCP-1] during MM.

	Estimate	Std. Error	*t*-value	Pr (>|t|)
(Intercept)	345.1181	60.3095	5.722	1.11e-05
TNF-α	0.5480	0.1463	3.747	0.0012
IL-10	0.0788	0.0641	1.229	0.2326
IP-10	0.0307	0.0164	1.875	0.0748
MCP-1	-0.2493	0.1644	-1.516	0.1444

The estimated coefficient (Estimate) corresponds to the fitted variable value, the standard error (Std. Error) indicates the variability of the estimated coefficient, the *t*-value measures whether or not the estimated coefficient for this variable is meaningful for the model, and the variable p-value (Pr (>|t|)) indicates the probability that the variable is not relevant in the model. R = 0.33 CI (-0.049–0.626); *p*-value = 0.1054.

**Table 7:** Multiple correlation analyses of [EPO + hemopexin + total heme] with [TNF-α + IL-10 + IP-10 + MCP-1] during NCM.

	Estimate	Std. Error	*t*-value	Pr (>|t|)
(Intercept)	203.0414	82.0068	2.476	0.0180
TNF-α	-0.0392	0.0977	-0.401	0.6910
IL-10	0.0532	0.0355	1.500	0.1420
IP-10	0.0132	0.0126	1.050	0.3010
MCP-1	0.0364	0.0378	0.962	0.3420

The estimated coefficient (Estimate) corresponds to the fitted variable value, the standard error (Std. Error) indicates the variability of the estimated coefficient, the *t*-value measures whether or not the estimated coefficient for this variable is meaningful for the model, and the variable p-value (Pr (>|t|)) indicates the probability that the variable is not relevant in the model. R = 0.50 CI (0.242–0.691); *p*-value = 0.0008.

**Table 8:** Multiple correlation analyses of [EPO + hemopexin + total heme] with [TNF-α + IL-10 + IP-10 + MCP-1] during CM.

	Estimate	Std. Error	*t*-value	Pr (>|t|)
(Intercept)	330.9000	60.8000	5.442	1.59e-06
TNF-α	-0.0001	0.1243	-0.001	0.9991
IL-10	0.0856	0.0296	2.894	0.0056
IP-10	-0.0198	0.0097	-2.038	0.0468
MCP-1	0.1017	0.0318	3.200	0.0024

The estimated coefficient (Estimate) corresponds to the fitted variable value, the standard error (Std. Error) indicates the variability of the estimated coefficient, the *t*-value measures whether or not the estimated coefficient for this variable is meaningful for the model, and the variable p-value (Pr (>|t|)) indicates the probability that the variable is not relevant in the model. R = 0.49 CI (0.264–0.665); *p*-value = 0.0002.

**Table 9:** Multiple correlation analyses of [EPO + hemopexin + total heme] with [TNF-α + IL-10 + IP-10 + MCP-1] for all *P*. *falciparum*-infected patients.

	Estimate	Std. Error	*t*-value	Pr (>|t|)
(Intercept)	320.3575	41.2283	7.770	3.2e-12
TNF-α	0.0278	0.0691	0.403	0.6879
IL-10	0.0616	0.0224	2.750	0.0069
IP-10	-0.0067	0.0071	-0.941	0.3487
MCP-1	0.0602	0.0252	2.390	0.0185

The estimated coefficient (Estimate) corresponds to the fitted variable value, the standard error (Std. Error) indicates the variability of the estimated coefficient, the *t*-value measures whether or not the estimated coefficient for this variable is meaningful for the model, and the variable p-value (Pr (>|t|)) indicates the probability that the variable is not relevant in the model. R = 0.28 CI (0.049–0.466); *p*-value = 0.0019.

**Fig 2:**
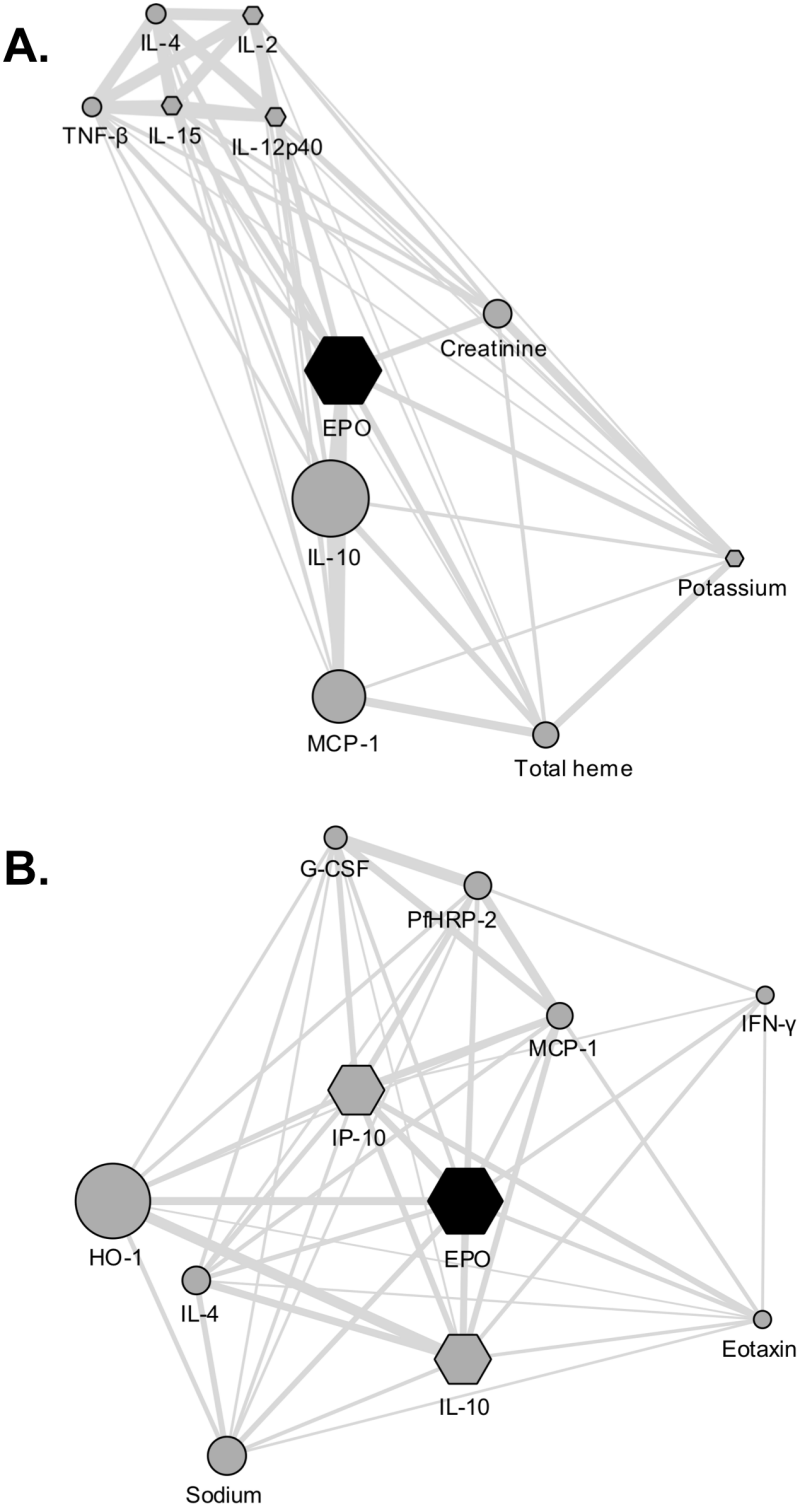
Sub-networks showing the ten parameters most related to plasma level of EPO during malaria. Sub-networks obtained for (A) the NCM group and the (B) CM group. Nodes with the highest number of connections with other parameters are represented by hexagons. For each parameter, the size of the node is associated with its level of correlation with EPO levels. Interactions between parameters are represented by edges (lines), thicker lines representing a higher confidence level for a given interaction.

Then, to complement our analyses, we used GeneMANIA, a plugin of the Cytoscape software that allows co-expression or co-regulation links to be identified between measurable objects [28]. This bioinformatics tool, classically used to generate co-expression networks from microarray data, was adapted here to the correlation analysis of quantifiable clinical, biological parameters and cytokine production correlating with disease severity in our *P*. *falciparum* infected Odisha patients. In particular, we sought to identify cytokines and bio-clinical parameters that correlated with EPO levels during CM but did not correlated with EPO levels in NCM malaria patients. In both groups, we found that the 10 parameters that most tightly correlated with EPO levels included IL-10 and MCP-1 (Fig 2), which confirms our multiple correlation analyses. However, it was striking to observe that EPO levels closely correlated with HO-1 levels in NCM patients (Fig 2A), while it correlated with heme during CM (Fig 2B).

## Discussion

In the light of divergences reported in the literature on circulating levels of EPO during human CM, we investigated EPO levels in a population of patients with distinct disease manifestations, epidemiology, environmental parameters and genetic background than those described earlier in the literature [10,11,16]. We found an increase in EPO levels in the plasma of *P*. *falciparum*-infected patients from Odisha with severe malaria, and particularly with fatal CM. It is noteworthy that a positive correlation of EPO levels with prolonged coma and survival was also reported earlier in Uganda, but only in patients with less than 8 g/dL hemoglobin [16]. As the destruction of red blood cells and the inhibition of erythropoiesis are known to trigger the production of EPO in response to hypoxia, it is conceivable that increased EPO levels observed in patients from Uganda were attributable to severe anemia and not to CM [33]. Moreover, our NCM and CM patients did not exhibit the same response to EPO, and we observed a correlation between HO-1 and EPO levels during NCM but not CM. We have previously reported that increased MIP-1α levels discriminate NCM from CM in Odisha [20], while MIP-1α was shown by others to inhibit the formation of erythroid progenitors [34]. Therefore, a bystander effect of MIP-1α on erythropoiesis may partly explain the lack of correlation between EPO and hemoglobin levels during NCM, reinforcing the idea that NCM and CM are two different disease phenotypes governed by distinct pathological mechanisms.

Previous studies suggest that EPO production increases with age, and within the normal range of hemoglobin [16,35]. Within our cohort, EPO levels were not affected by age, neither in control nor in infected patients in Odisha (S1 Fig). As most of the reported studies were conducted in children, further analysis in younger Odissi individuals would be necessary to better explain the contradictory results between the different populations studied. One can note that the discrepancy due to age may also reflect the level of clinical immunity, which is known to develop with time under constant exposition to the parasite according to the endemicity of *P falciparum* [36]. While Kenya and Mozambique are hyperendemic for *P*. *falciparum*, Odisha is mesoendemic with a transmission once a year peaking during the rainy season.

Other than age, distinct environmental determinants such as the genetic background and hematological parameters may also influence the pathophysiology of CM, and consequently the role of EPO in the outcome of malaria. In fact, molecules released during parasite multiplication within red blood cells, such as heme, may contribute to increased EPO levels during malaria [32]. We have previously described a rise in heme levels with malaria severity and, particularly during CM, in the same group of patients [21]. In the same line, Spearman’s and multivariate analyses have shown that EPO, total heme and hemopexin levels were significantly correlated during CM. Given our findings, we propose that the release of these endogenous molecules up-regulates EPO levels. Nevertheless, the correlation observed between HO-1 and EPO levels during NCM, but not CM, suggests a heme-dependent amplification loop of EPO that may be linked to the degradation of heme by HO-1. In addition, the significant multivariate correlation of EPO, hemopexin and total heme with TNF-α, IL-10, IP-10 and MCP-1 suggests the implication of EPO in the complex network of factors of the immune system influenced by heme during severe malaria. Free heme is more likely to induce a pro-inflammatory response during acute malarial infection, and, similarly to EPO, we have previously reported a positive correlation between heme and TNF-α, IL-10, IP-10 and MCP-1 during *P*. *falciparum* malaria in India [21]. Unlike IL-10 that is protective against CM, TNF-α, IP-10 and MCP-1 were shown to be important mediators in the induction of rodent and human CM [20,37–39]. However, it is unclear whether high levels of EPO are contributing to, or are insufficient in preventing inflammation associated to CM. In fact, EPO is likely to affect the production of cytokines by immune cells but the overall response is still not clear. For instance, EPO mediates the pro-inflammatory response of macrophages and dendritic cells stimulated with lipopolysaccharide *in vitro*, as suggested by their increased production of IL-12 and decreased production of IL-10 [40,41]. However, Nairz et al. have reported a decreased production of TNF-α by lipopolysaccharide-stimulated primary macrophages that were pretreated with EPO [42]. In the line with these observations, exogenous EPO was shown to decrease the expression of pro-inflammatory cytokines in the brain of C57BL/6 and CBA/J mice infected with *P*. *berghei* ANKA [12,13].

Rodent models are essential for understanding the pathophysiology of malaria. However, they are insufficient as proof of principle for the development of human therapeutic targets. Our analysis suggests that, in contrast to what was reported earlier from Sub-Saharan Africa, EPO levels increased in CM patients from Odisha. These observations suggest differences in the pathogenesis of malaria between Sub-Saharan African and South Asian populations and highlight the need to better evaluate malaria-related parameters in different geographical and epidemiological contexts for the optimization of anti-malarial and adjunctive treatments.

## Supporting Information

S1 Fig Spearman rank’s correlation analyzes of plasma EPO levels with age in different clinical groups of malaria-infected patients. (PDF)
