# Supplementary material for: Erythropoietin Levels Increase during Cerebral Malaria and Correlate with Heme, Interleukin-10 and Tumor Necrosis Factor-Alpha in India
Source: PLoS One. 2016 Jul 21;11(7):e0158420. doi: 10.1371/journal.pone.0158420 (PMC4956275; doi:10.1371/journal.pone.0158420)
Supplement: S1 Fig — (PDF) [file pone.0158420.s001.pdf]

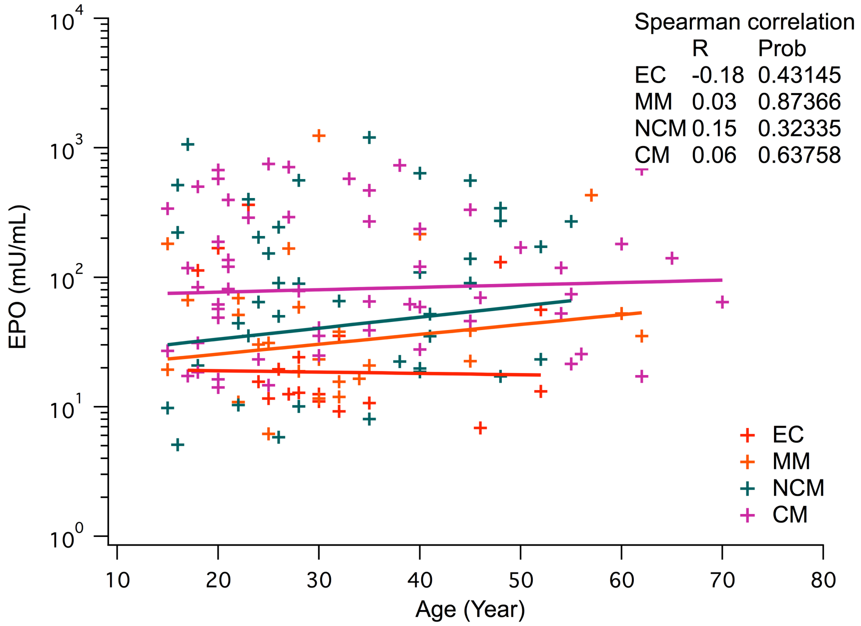

S1 Figure. Spearman rank's correlation analyzes of plasma EPO levels with age in different clinical groups of malaria-infected patients.
